# Clinical and Virological Profiles Associated with CINTEC^®^ PLUS Positivity: A Data-Driven Clustering and Modeling Study

**DOI:** 10.3390/diagnostics15172200

**Published:** 2025-08-29

**Authors:** Iulian-Valentin Munteanu, Demetra Socolov, Razvan Socolov, Ana-Maria Adam, Gigi Adam, Ingrid-Andrada Vasilache, Petronela Vicoveanu, Valeriu Harabor, Anamaria Harabor, Alina-Mihaela Calin

**Affiliations:** 1 Clinical and Surgical Department, Faculty of Medicine and Pharmacy, ‘Dunarea de Jos’ University, 800216 Galati, Romania; 2 Department of Mother and Child Care, “Grigore T. Popa” University of Medicine and Pharmacy Iasi, 700115 Iasi, Romania; 3 Department of Pharmaceutical Sciences, Faculty of Medicine and Pharmacy, ‘Dunarea de Jos’ University, 800216 Galati, Romania; 4 Department of Mother and Newborn Care, Faculty of Medicine and Biological Sciences, ‘Ștefan cel Mare’ University, 720229 Suceava, Romania

**Keywords:** dual-stain cytology, CINtec^®^ PLUS positivity, XGBoost, modeling, clinical risk profile, clusters

## Abstract

**Background/Objectives**: The diagnostic performance of CINtec^®^ PLUS can be influenced by numerous patient characteristics and risk factors. The aim of this retrospective study was to evaluate and model the risk factors associated with CINtec^®^ PLUS test positivity in patients undergoing cervical cancer screening and to assess their predictive performance for the prediction of cervical intraepithelial neoplasia (CIN) 2/3 using an unsupervised machine learning-based model. **Methods**: Medical data of 134 patients with human papillomavirus (HPV) infection who underwent CINtec^®^ PLUS testing were used to model the impact of risk factors on dual-stain cytology positivity and to evaluate the predictive performance for CIN2/3. **Results**: The gradient boosting classifier for the prediction of CINtec^®^ PLUS positivity using clinical risk factors had a precision of 75%, an overall accuracy of 0.62, and an area under the curve (AUC) value of 0.77. Body mass index and age were the most important variables in this model. HSIL, ASC-US, and other high-risk HPV strains increased the likelihood of a positive outcome. Overall AUC values for a positive test alone were 0.74 and 0.69 for CIN2 and CIN3 prediction, respectively. For CIN2 prediction, the XGBoost model performed well, with 71% sensitivity, 85% specificity, and an AUC value of 0.90. However, the model had 96% sensitivity, 25% specificity, and 0.58 AUC for CIN3 prediction. **Conclusions**: Patient characteristics and risk factors can influence CINtec^®^ PLUS positivity rates and they need to be carefully considered before choosing a specific management.

## 1. Introduction

In recent years, CINtec^®^ PLUS test (p16/Ki-67 dual stain) has gained international attention for its capabilities and risk stratification of cervical dysplasia. This test is based on complex molecular mechanisms that are altered during cervical carcinogenesis, and numerous factors can influence the degree of gene expression.

In oncogenic human papillomavirus (HPV) infections, the viral oncoproteins E6 and E7 disrupt p53 and Rb proteins’ tumor suppressor activities, causing uncontrolled cell proliferation [1,2]. HPV E7 inactivates Rb, releasing E2F transcription factors that advance the cell cycle [3]. The CDKN2A gene on 9p21 encodes the tumor suppressor protein p16INK4a [4]. It regulates the cell cycle by suppressing CDK4/6, which are necessary for the G1–S cell cycle transition [5]. Since CDK4/6 activity is deregulated, p16INK4a is overexpressed to compensate, indicating HPV-mediated oncogenic transformation [6]. On the other hand, protein Ki67 is present during G1, S, G2, and mitosis and absent in G0 [7], signaling active cell proliferation.

In March 2020, the p16ink4a/Ki-67 dual stain became commercially available as an additional tool of triage of patients with positive HPV results [8]. By 2024, the Enduring Consensus Cervical Cancer Screening and Management Guidelines Committee issued specific recommendations for using the CINtec^®^ PLUS test to guide management [9]. Specifically, if both tests (CINtec^®^ PLUS and HPV genotyping) are positive, immediate colposcopy is recommended. If the CINtec^®^ PLUS test is negative, the recommendation is to repeat HPV testing in one year, unless the person tests positive for HPV16 or HPV18 or has high-grade abnormalities on a Pap smear, which automatically indicate colposcopy examination [9].

Current evidence from systematic reviews of the literature indicates that dual-stain cytology has higher sensitivity than cytology, as well as higher specificity than HPV genotyping [10]. Moreover, the pooled sensitivity of dual-stain cytology was 88%, while the pooled specificity was 58% in a recent quantitative meta-analysis [11]. Peteers and colleagues demonstrated that dual-stain cytology is more specific for the diagnosis of CIN2+ (59–82% versus 23–53%) and CIN3+ (36–76% versus 19–56%) than high-risk HPV genotyping in patients with minor abnormalities [12]. The most recent systematic review indicated that dual-stain cytology was correlated with a more severe histological diagnosis and with HPV positivity [13].

The diagnostic and/or predictive performance of CINtec^®^ PLUS can be influenced by numerous patient characteristics and risk factors, such as age, previous history of abnormal cytology, type of HPV strain, behavioral profile, etc. [14,15,16]. Thus, it is important to evaluate specific patient profiles and to integrate all medical data before deciding on a specific approach. In this context, recent literature data outlined the potential of machine learning approaches for superior capabilities of stratification of patients at risk of cervical cancer [17,18,19,20].

As far as we know, literature data are very scarce regarding the use of machine learning (ML)-based models for the prediction of cervical intraepithelial neoplasia, and most of the studies focus on the prediction of cervical cancer. For example, Hariprasad et al. used the extreme gradient boosting (XGBoost) classifier to predict the risk of cervical cancer based on 36 clinical features of 858 patients from Venezuela and demonstrated an overall accuracy of the model of 98.9% [21]. In this study, the most important features were related to HPV positivity, smoking status, and personal history of sexually transmitted disorders. Another study tested six classification algorithms, including an artificial neural network, a Bayesian network, a support vector machine (SVM), a random tree, a logistic tree, and an XGBoost tree for the detection of early cervical cancer and demonstrated that the maximum accuracy of 94.94% was achieved by the XGBoost model [22]. The authors outlined the potential of these models to outperform traditional statistical models in terms of analyzing complex data and uncovering prognostic features.

Moreover, Zhai et al. conducted a retrospective analysis to evaluate residual or recurrent high-grade CIN (CIN2 or worse) after a loop electrosurgical excision procedure (LEEP) during follow-up using eight machine learning models. The authors demonstrated that the XGBoost algorithm had the highest accuracy (81.3%) and the top three features were represented by margin status, CIN degree, and HPV status [23].

Thus, the primary aim of this retrospective study was to evaluate and model the clinical, behavioral, and virological factors associated with CINtec^®^ PLUS test positivity in patients undergoing cervical cancer screening and to assess their predictive performance for the prediction of CIN2 and CIN3 using an unsupervised machine learning-based model.

## 2. Materials and Methods

Persistent infection with high-risk human papillomavirus is the necessary condition of cervical carcinogenesis, but HPV positivity alone has limited specificity for high-grade disease, creating a need for secondary triage tools that better distinguish patients at risk for CIN2+ from those likely to regress. Dual-stain CINtec^®^ PLUS (p16/Ki-67) has emerged as a promising triage test with higher sensitivity than cytology and improved specificity compared with HPV genotyping alone, but its performance in real-world, opportunistic screening settings in Eastern Europe remains underreported. Moreover, the influence of patient characteristics on CINtec^®^ PLUS positivity and downstream risk of CIN2/3 is not well defined in this population.

The primary hypothesis for this study was that CINtec^®^ PLUS positivity is independently associated with hrHPV status and high-risk clinical features.

Secondary hypotheses:

(i) A machine learning-based model integrating clinical, behavioral, virological, and cytologic factors outperforms CINtec^®^ PLUS alone for predicting CIN2/3;
(ii) SHAP-based feature importance will highlight the clinical risk factors that have major importance for the prediction of CINtec^®^ PLUS positivity;
(iii) UMAP + DBSCAN will identify distinct patient clusters with differential CINtec^®^ PLUS positivity and histologic outcomes.

We conducted a retrospective cohort study of patients with HPV infection who underwent self-referred or targeted cervical cancer screening in association with CINtec^®^ PLUS. Participants were recruited from colposcopy clinics across the Moldavian region, Romania, between 2023 and 2025. Approval for this study was obtained from the Institutional Ethics Committee of Clinical Hospital of Obstetrics and Gynecology “Buna vestire” Galati (No. 115/05.01.2021). All participants provided written informed consent.

We collected data from patients who had at least one baseline evaluation with cervical cytology, HPV genotyping, and/or histopathology, with a CINtec^®^ PLUS result. The exclusion criteria were prior cervical cancer, immunosuppression, hysterectomy, pregnancy, or incomplete medical records.

We collected the following types of information from the patients’ medical records: demographic data, body mass index (BMI), smoking status, number of sexual partners, age at sexual debut, history of combined oral contraceptive (COC) use, history of sexually transmitted infections (STIs), reproductive and gynecological history, and HPV vaccination status. Virological data included HPV genotyping results, specifically the presence of HPV 16/18, other high-risk HPV types, and low-risk HPV types.

We also extracted diagnostic test outcomes, such as the result of the CINtec^®^ PLUS dual staining test (Roche mtm Laboratories AG, Mannheim, Germany) and related cytological findings. Cytological classifications were documented as LSIL (low-grade squamous intraepithelial lesion), HSIL (high-grade lesion), ASC-US (atypical squamous cells of undetermined significance), and NILM (negative for intraepithelial lesion or malignancy) [24]. Finally, reference diagnostic endpoints such as histologically confirmed CIN2 or CIN3 (used as gold standard outcomes) were also recorded for comparative and modeling purposes.

We used a binary logistic regression for quantifying the impact of various predictors over a positive CINtec^®^ PLUS test result, and we reported odds ratios (OR) and 95% confidence intervals (CI). We also assessed the discrimination of the logistic model using receiver operating characteristic (ROC) analysis, and significance was defined at *p* < 0.05.

The predicted probability of CINtec^®^ PLUS positivity from the logistic model was used to create a clinical risk score, scaled from 0 to 10. The scores were rounded to the nearest integer and used to stratify patients into 3 risk categories: low risk (score 0–3), intermediate risk (score 4–6), and high risk (score 7–10). ANOVA analysis and a post hoc Bonferroni test were used to assess the differences in the calculated risk scores depending on the risk category.

We further created a gradient boosting classifier using the XGBoost (eXtreme gradient boosting) framework for the prediction of CINtec^®^ PLUS positivity using clinical risk factors. The model was trained using a stratified 70/30 train–test split. Model calibration and discrimination were evaluated with ROC analysis, and we reported the precision, recall, F1 score, and area under the receiver operating characteristic (ROC) curve (AUC).

In order to evaluate the feature importance and interpret the model’s decisions, we applied SHapley Additive exPlanations (SHAP) and reported SHAP summary plots that ranked predictors based on their mean absolute SHAP values.

For unsupervised patient segmentation, we applied the density-based spatial clustering of applications with noise (DBSCAN) algorithm, following dimensionality reduction using uniform manifold approximation and projection (UMAP). UMAP was performed on standardized predictor variables to obtain a two-dimensional embedding. DBSCAN-defined clusters were visualized on the UMAP projection to assess phenotypic heterogeneity.

Hex-binned heatmaps were created for each feature to further investigate the distribution of clinical and behavioral variables within the low-dimensional UMAP space. This method facilitated the visualization of localized trends in feature distribution among clusters.

We also conducted a predictive analysis for histologically confirmed cervical intraepithelial neoplasia grades 2 and 3 based on histopathological confirmation for evaluating the diagnostic performance of CINtec^®^ PLUS and to compare it to the XGBoost model. The model included clinical and behavioral predictors such as age, BMI, smoking status, sexual behavior, contraceptive use, HPV status, and cytological findings. Data preprocessing included standardization of continuous variables and oversampling of the minority class using the synthetic minority over-sampling technique (SMOTE).

Model training was conducted using a stratified 70/30 train–test split. Performance evaluation on the test set included the same diagnostic metrics described above.

All analyses were performed in Python (version 3.11.13, Python Software Foundation, Wilmington, DE, USA) using the scikit-learn and XGBoost libraries, as well as STATA SE (version 19.5, StataCorp, College Station, TX, USA). Statistical significance was defined at *p* < 0.05.

## 3. Results

A total of 134 medical records were included in the analysis, which were divided by the results of the CINtec^®^ PLUS test: group 1 (63 patients with negative test result) and group 2 (71 patients with positive test result). Our univariate analysis indicated that patients with a positive test result had significantly higher BMI (*p* = 0.001), along with a significantly higher prevalence of smoking history (*p* = 0.001) and HSIL (*p* < 0.001) (Table 1).

**Figure 1:**
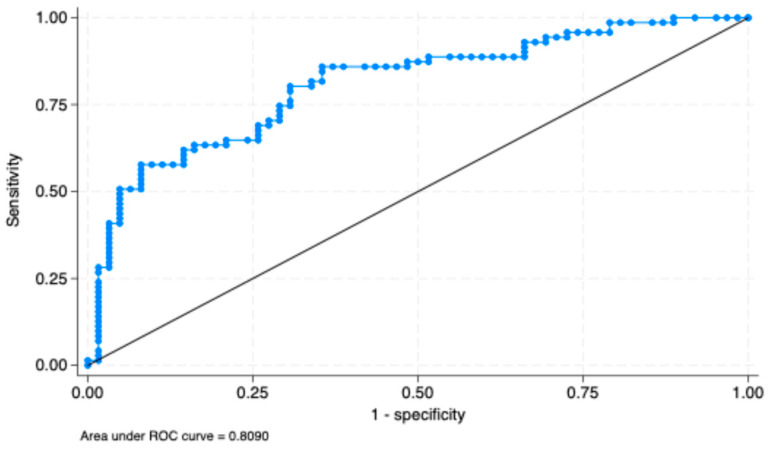
ROC curve for the prediction of a positive CINtec^®^ PLUS test using the logistic regression model.

In Table 2 are presented the results from the logistic regression model that quantified the impact of various predictors over a positive CINTEC^®^ PLUS test result. Our data indicated that age (OR: 1.058, 95%CI: 1.006–1.111, *p* = 0.027), BMI (OR: 1.829, 95%CI: 0.729–2.942, *p* = 0.004), positive smoking history (OR: 1.241, 95%CI: 0.079–2.735, *p*= 0.012), and HSIL cytology (OR: 7.06, 95%CI: 2.287–21.836, *p* = 0.001) significantly increased the odds of a positive test result. The overall AUC value of the model for the prediction of a positive test result was 0.809 (Figure 1).

**Figure 2:**
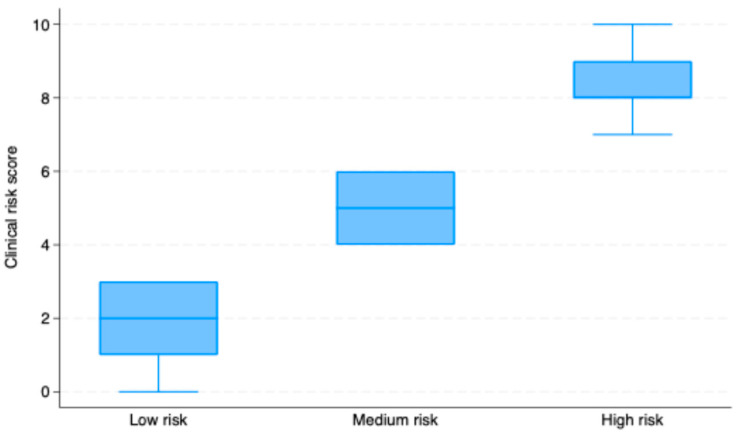
Boxplot demonstrating the mean calculated risk scores for a positive CINTEC^®^ PLUS test result depending on the risk category.

Table 3 and Figure 2 present the differences in the calculated risk scores for a positive CINTtec® PLUS test result across three different risk groups: low, medium, and high. Our data indicated that patients classified as low risk had a mean score of 2.03 (SD = 0.94), while those in the medium risk group had a significantly higher mean score of 4.98 (SD = 0.79). The high-risk group exhibited the highest average score, with a mean score of 8.38 (SD = 0.99). The ANOVA analysis demonstrated a statistically significant difference in mean clinical scores among the three groups (*p* < 0.001). Post hoc comparisons using Bonferroni correction further revealed that all pairwise differences were statistically significant (*p* < 0.001).

**Figure 3:**
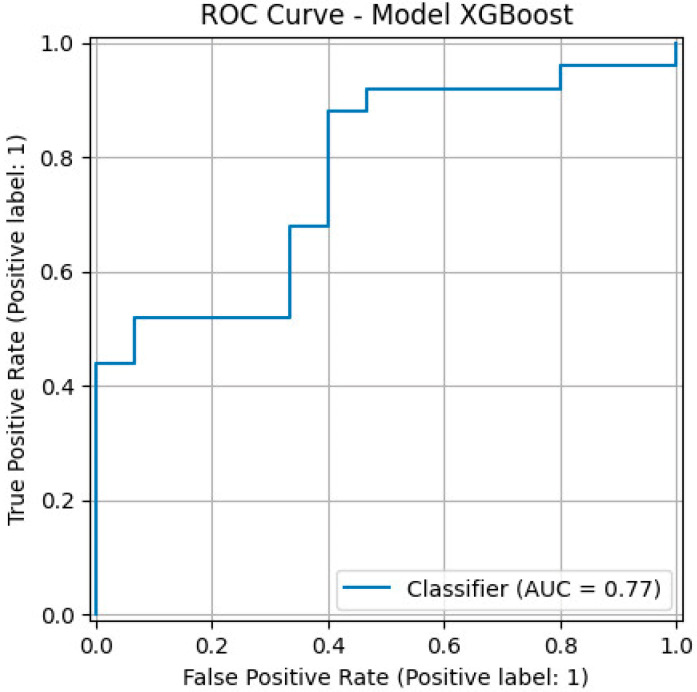
ROC curve for XGBoost model for the prediction of a positive CINTEC^®^ PLUS test result based on risk factors.

The gradient boosting classifier for the prediction of CINtec^®^ PLUS positivity using clinical risk factors (Figure 3) had a precision of 75%, a recall of 0.60, an F1 score of 0.67, an overall accuracy of 0.62, and an AUC value of 0.77.

**Figure 4:**
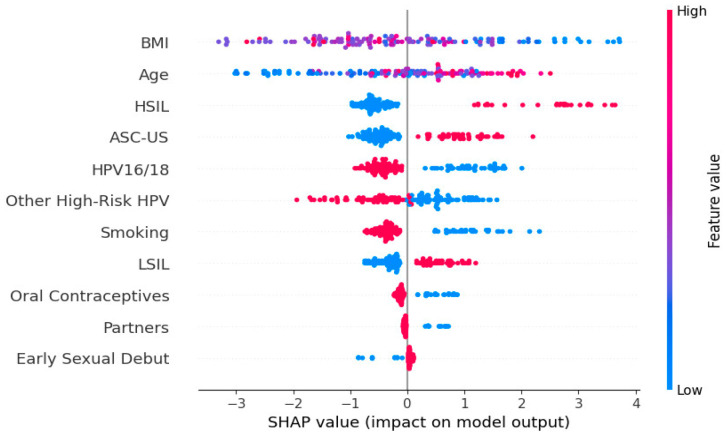
SHAP summary plot displaying the impact of individual features on the model output for predicting CINtec^®^ PLUS test positivity.

**Figure 5:**
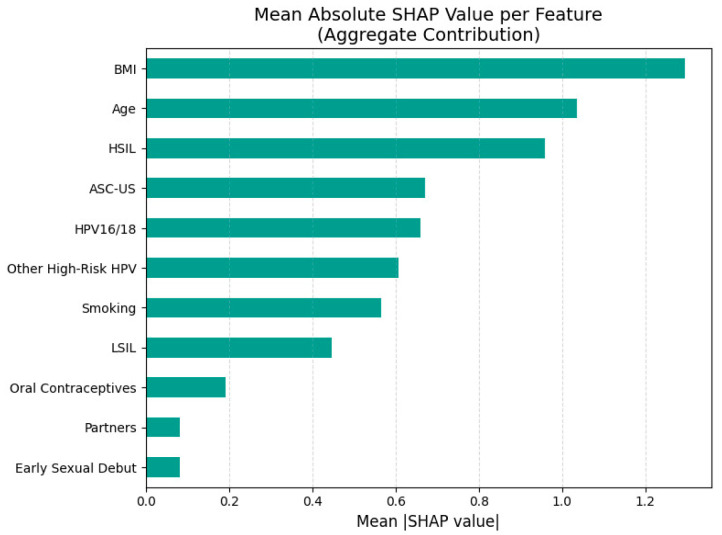
Aggregate feature importance based on mean absolute SHAP values for CINtec^®^ PLUS positivity prediction.

While the XGBoost model achieved high predictive accuracy for CINtec^®^ PLUS positivity, machine learning outputs are often criticized for their “black box” nature, limiting their acceptance in clinical practice. Explainable AI methods such as SHAP address this limitation by quantifying the contribution of each predictor to the model’s decision, both at a global and individual level. The SHAP summary plot illustrates the relative contribution and direction of influence for each predictor variable in the XGBoost model used to predict CINtec^®^ PLUS test positivity (Figure 4). Figure 5 represents the aggregate feature importance based on mean absolute SHAP values for CINtec^®^ PLUS positivity prediction.

The most important predictors in this model were BMI and age, with both higher and lower values associated with varying directions of impact on the model output. Higher BMI was generally associated with lower predicted probability (leftward SHAP values) of a positive test, suggesting a potential inverse association, while higher age appeared to increase the risk.

The presence of HSIL, ASC-US and other high-risk HPV types was generally associated with positive SHAP values, indicating that these variables increased the model’s predicted likelihood of a CINtec^®^ PLUS positive result. Smoking had a mostly positive impact on CINtec^®^ PLUS positivity when present, aligning with known risk factors for cervical dysplasia. The presence of HPV16/18 was generally associated with increased model output (positive prediction) but with less consistency than expected.

LSIL presence was associated with increased CINtec^®^ PLUS positivity. Behavioral and reproductive factors such as number of partners, use of oral contraceptives, and early sexual debut contributed modestly, and the direction of their effects varied.

**Figure 6:**
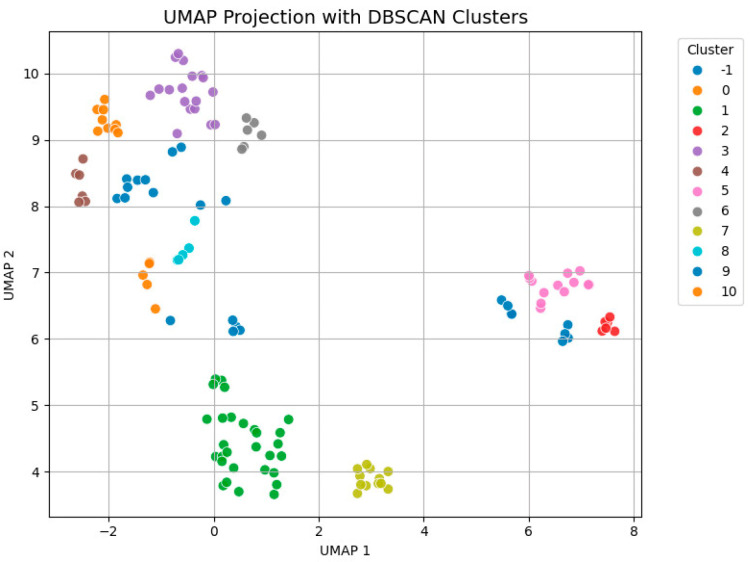
Patient segmentation using UMAP and DBSCAN clustering.

**Figure 7:**
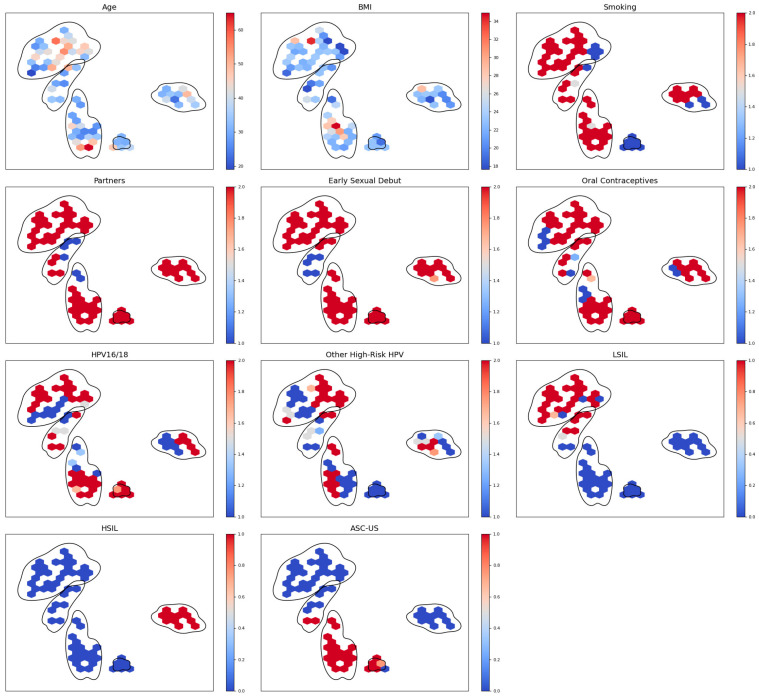
Hex-binned UMAP feature distribution plot.

Figure 6 illustrates the results of unsupervised clustering applied to standardized patient-level data using DBSCAN following dimensionality reduction via UMAP. Each point represents an individual patient, and its position in the 2-dimensional UMAP space reflects similarity in clinical, behavioral, cytological, and virological features. Figure 7 represents the hex-binned UMAP feature distribution plot.

The DBSCAN algorithm identified 11 distinct clusters (Clusters 0–10), in addition to a group of outliers labeled as Cluster −1, which represented patients who could not be assigned to any of the main clusters due to insufficient local density.

The dominant clusters were 1 (n = 29 patients), 3 (n = 18 patients), −1 (n = 17 patients), 5 (n = 13 patients), and 7 (n = 12 patients), which account for 66.4% of the total sample. Patients included in cluster −1 had a mean age of 34.4 years and a mean BMI of 22.9 kg/m^2^, with intermediate levels of smoking and early sexual debut, as well as the highest prevalence of HPV16/18 and high-grade cytological abnormalities.

Cluster 1 comprised slightly older patients (36.5 years) with elevated BMI (25.3 kg/m^2^) and high-risk behaviors, including smoking and multiple sexual partners. Nearly all had HPV16/18 infection.

Cluster 3 comprised the oldest patients (44.3 years), with a moderate BMI (24.6 kg/m^2^) and high exposure to smoking and sexual risk factors. There was high prevalence of both HPV16/18 and other high-risk HPV types, but none had HSIL.

Patients in cluster 5 had a mean age of 36.2 years and a normal BMI, with uniform high behavioral risk exposure, and a relatively low HPV16/18 prevalence. Patients in cluster 7 had a mean age of 32.8 years and low BMI, lacked HSIL, but showed elevated exposure to other high-risk HPV types.

**Figure 8:**
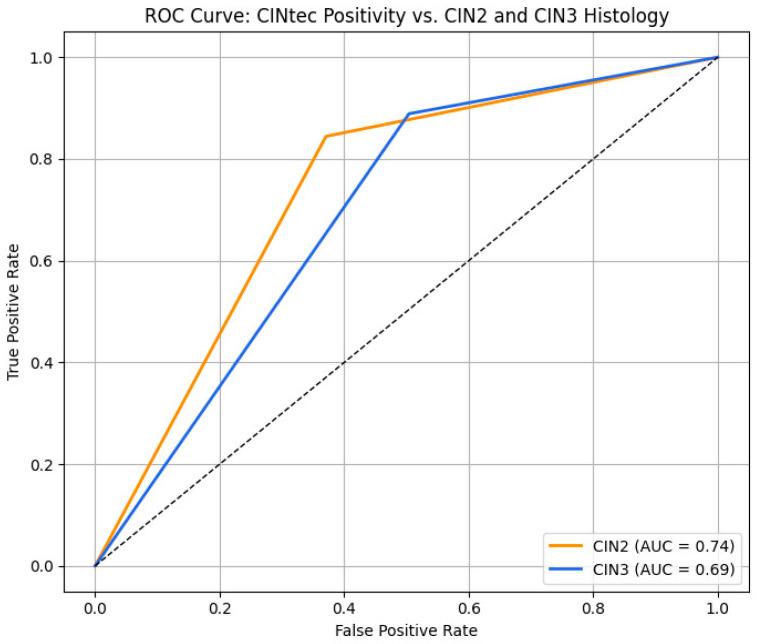
ROC curve for the prediction of CIN2 and CIN3 using a Positive CINtec^®^ PLUS result.

Table 4 and Figure 8 outline the results from ROC analysis that evaluated the predictive performance of a positive CINtec^®^ PLUS for the prediction of CIN2 and CIN3. Our results indicated that a positive test alone had overall accuracies for the prediction of CIN2 of 70% and of CIN3 of 52%. The overall AUC values were 0.74 for the prediction of CIN2 and 0.69 for the prediction of CIN3.

The same trend was observed when we tested the overall performance of the XGBoost model for CIN2 and CIN3 prediction. Thus, for CIN2 prediction, the model achieved superior performance, with a sensitivity of 71% and a specificity of 85%. The overall accuracy reached 0.80, and the AUC was 0.90.

For CIN3, the model’s sensitivity was high (96%), but the specificity was low (25%). Despite this, the model maintained a high F1 score (0.92) and accuracy (0.85), although the AUC was 0.58.

## 4. Discussion

In this retrospective study, we evaluated the clinical, behavioral, and virological factors associated with CINtec^®^ PLUS positivity and we examined the predictive performance of a machine learning model for the detection of histologically confirmed CIN2 and CIN3. Our results highlighted the risk factors that were significantly associated with CINtec^®^ PLUS positivity and created risk scores based on the logistic regression model that incorporated relevant risk factors identified in our cohort of patients. Moreover, we outlined significant clusters of patient phenotypes that were associated with an increased likelihood of CINtec^®^ PLUS positivity and could orient the clinical management of these patients.

We found that high-grade cytological abnormalities, particularly HSIL, positive smoking history, older age, and BMI were independent risk factors associated with CINtec^®^ PLUS positivity, and the logistic regression model that included these variables achieved an AUC value of 0.80, which indicated high predictive performance. Also, patients who had multiple risk factors had an increased risk score for CINtec^®^ PLUS sensitivity. When we employed the XGBoost model to predict CINtec^®^ PLUS positivity, we achieved a precision of 75%, an overall accuracy of 0.62, and an area under the curve (AUC) value of 0.77.

Previous literature data indicated that p16 levels are increased in older patients as they indicate cellular senescence, thus older age could be an important covariate that modulates the positivity of the evaluated test [15,25,26]. Moreover, smoking appears to have a significant impact in the positivity of CINtec^®^ PLUS, as demonstrated by a prospective longitudinal study conducted by White et al., which included 275 HPV-positive patients who presented for colposcopy examination for cytological abnormalities (LSIL/ASCUS). The authors demonstrated that patients with nicotine metabolite concentrations greater 500 ng/mL had an increased risk of positive test (OR: 1.678; 95%CI: 1.027–2.740) and CIN2+ and CIN3+ (OR: 1.816; 95%CI: 1.107–2.977 and OR: 2.453; 95%CI: 1.200–5.013, respectively) in comparison to non-smokers. Moreover, they showed that patients with a positive test who smoked had an increased risk of CIN2+ and CIN3+ (OR: 2.290; 95%CI: 1.017–5.159 and OR: 3.506; 95%CI: 1.534–8.017, respectively) [27].

Trzeszcz et al. evaluated the performance of three models: primary HPV genotyping with CINtec^®^ PLUS triage, primary cytology with reflex HPV genotyping, and primary cytology alone for the detection of HSIL-risk in patients under 30 years old [28]. The authors demonstrated that the first model had significantly higher sensitivity for the detection of HSIL, quantified with CIN2 or worse, than the other two models (83.3% vs. 70.8%/45.8%) and had significantly higher positive and negative predictive values (PPV:29.4%/21.3%/22.9%; NPV:91.7%/82.9%/82.2%). Also, additional literature data confirmed the use of CINtec^®^ PLUS for the detection of HSIL lesions [29,30].

Tóth et al. conducted a retrospective analysis of 395 patients undergoing specific excisional treatment that evaluated the association between inflammatory markers, such as neutrophil/lymphocyte ratio (NLR), platelet/lymphocyte ratio (PLR), and lymphocyte/monocyte ratio (LMR), and p16 positivity in CIN cases [31]. They showed that increased levels of NLR were significantly associated with p16 positivity (*p* = 0.011) and HPV positivity (*p* = 0.04), which could outline the impact of systemic inflammation in cervical carcinogenesis.

Our machine learning model (XGBoost) demonstrated high accuracy and predictive power for CIN2 (AUC = 0.90) and outperformed the CINtec^®^ PLUS test alone in terms of overall performance. These results are supported by literature studies that highlight the performance of machine learning algorithms in interpreting complex screening and diagnostic models for early detection of high-grade lesions and cervical cancer [32,33]. However, for CIN3 prediction, although sensitivity remained high (96%), specificity was low (25%), which limits its stand-alone diagnostic utility for this more severe outcome. Dual-stain cytology achieved high sensitivity but low to moderate specificity for both evaluated outcomes.

Literature data indicated that dual-stain cytology demonstrated superior performance compared to cytology in triaging high-risk HPV-positive patients, offering higher sensitivity, specificity, and overall diagnostic accuracy for detecting both CIN2+ and CIN3+ lesions. However, while the sensitivity was high, the specificity was moderate-low. A retrospective analysis that included 1130 patients compared the performance of p16/Ki67 immunostaining to that of cytology alone for the detection of high-grade cervical dysplasia (CIN2+/CIN3+) [28]. The authors showed that in HPV16/18-positive patients, dual-stain triage was significantly more specific than cytology for both CIN2+ (53.1% vs. 16.8%) and CIN3+ (45.9% vs. 17.0%), with comparable sensitivity (CIN2+: 95.7% vs. 84.8%; CIN3+: 100.0% vs. 87.5%). In patients with other high-risk HPV types, dual-stain cytology had significantly higher specificity (CIN2+: 51.3% vs. 15.3%; CIN3+: 44.5% vs. 16.5%) while maintaining high sensitivity (CIN2+: 92.3% vs. 74.4%; CIN3+: 90.9% vs. 81.8%).

A cross-sectional study conducted in China included 10,500 patients who underwent the ThinPrep cytologic test (TCT) and p16/Ki-67 dual-stain test and compared the performance of these two methods for the triage of high-risk HPV-positive patients [34]. The authors found out that the p16/Ki-67 dual-stain test showed significantly better performance than traditional cytology in detecting ≥CIN2 lesions, with higher sensitivity (82.8% vs. 66.7%), specificity (51.6% vs. 44.4%), PPV (33.2% vs. 25.8%), NPV (91.2% vs. 82.1%), and overall accuracy (58.6% vs. 49.4%). Similar performance was observed for ≥CIN3 detection, where the dual-stain test again outperformed cytology across all metrics, including sensitivity (89.5%), specificity (47.2%), PPV (14.7%), and NPV (97.8%).

A predictive modeling study that included data from 267 healthcare centers in China that participated in a national cervical cancer screening project compared and validated four machine learning models: XGBoost, support vector machine (SVM), random forest (RF), and naïve Bayes (NB) for CIN2+ and CIN3+ prediction [35]. The authors showed that, in external validation, XGBoost demonstrated consistently high sensitivity for CIN2+ detection, ranging from 85.6% to 100%, while specificity varied notably between cohorts, with values ranging from 34.7% to 98.1%. Moreover, the AUROC values for CIN2+ ranged between 0.781 and 0.989, and accuracy ranged from 36.3% to 97.9%, with higher values observed in cohorts with more balanced disease prevalence and lower false-positive rates.

Also, for CIN3+, XGBoost maintained very high sensitivity, ranging from 90.2% to 100%, while its specificity showed wide variability, from 34.6% to 97.6%. AUROC values for CIN3+ remained high across external cohorts, ranging from 0.793 to 0.944, and the corresponding accuracy values ranged between 35.1% and 97.6% [35].

Akter et al. utilized three machine learning models, decision tree (DT), RF, and XGBoost, to predict cervical cancer risk using behavioral and feature data and demonstrated substantial performance enhancements compared to current methodologies, attaining an accuracy of 93.33% [36]. Uddin et al. tested ten different machine learning models on a public dataset that comprised 858 patients in order to predict cervical cancer based on clinical risk factors such as cytology, HPV strain, sexually transmitted infections, etc. [37]. The authors showed that their best-performing model, utilizing hard voting with a combination of multilayer perceptron (MLP), RF, XGBoost, and principal component analysis (PCA), achieved an accuracy of 99.19% and 100% sensitivity for cervical cancer prediction.

Furthermore, our application of unsupervised clustering (UMAP + DBSCAN) revealed distinct patient subgroups with meaningful phenotypic patterns. Cluster analysis outlined heterogeneous profiles based on behavioral and virological features. Similar unsupervised approaches have been employed in oncology and epidemiology to highlight specific phenotypic profiles within disease populations, suggesting that such methods may enhance patient stratification in cervical screening programs [38,39,40]. There is a lack of literature data regarding the use of unsupervised clustering for identifying phenotypic patterns of patients with cervical dysplasia. However, a recent study demonstrated that several depression-associated genes display mutation patterns across different cancer types, including skin, uterine, cervical, stomach, and prostate cancers [41]. The authors used clustering analyses with HJ biplot K-means and DBSCAN and identified groups of genetic variants linking depressive traits with oncogenic mutations, suggesting shared biological pathways between these conditions. Machine learning models were further applied to classify cancers with overlapping genetic signatures related to depression. Among the models tested, random forest, KNN, and neural networks achieved the strongest performance, reaching F1 scores of 0.95 or higher. Another study evaluated MnM, a machine learning-based tool, to disentangle single-cell replication timing profiles from heterogeneous samples [42]. This approach provided the ability to separate somatic copy number alterations from copy number variations attributable to DNA replication processes, enabling the investigation of replication timing diversity and chromosomal aberrations across different cancer contexts. Finally, a pilot study examined the use of machine learning methods for the automated diagnosis and staging of cervical cancer based on questionnaire-style data. The results indicated that the naïve Bayes classifier demonstrated strong predictive performance, with classification accuracies exceeding 90% across multiple evaluation metrics, making it suitable for identifying the presence of cervical cancer and the extent of disease [43].

The strengths of this study include the integration of histopathologically confirmed endpoints, the use of machine learning approaches for prediction purposes, and the application of unsupervised clustering for defining patient phenotype in the evaluated cohort. However, there are several inherent limitations of this study that should be taken into account. First, the retrospective design may be subject to inherent selection and information biases. Second, the relatively sample size could limit the statistical power to detect more subtle associations, assessment of its predictive value for disease recurrence, or long-term risk stratification.

Future research directions could evaluate whether incorporating cluster assignment from unsupervised analysis into XGBoost models improves the prediction of CIN2+ and CIN3+. This assessment could determine if patient-level phenotypes, derived from multidimensional clinical, behavioral, and virological profiles, provide additional predictive value.

## 5. Conclusions

This study indicates that machine learning, particularly the XGBoost model, effectively predicts CINtec^®^ PLUS positivity and histologically confirmed CIN2 lesions by utilizing clinical, behavioral, and virological risk factors. The model demonstrated strong overall performance in predicting CINtec^®^ PLUS, with age and BMI identified as significant predictors. High-grade cytological abnormalities, a history of smoking, and HPV16/18 infection significantly increased the likelihood of a positive test result.

XGBoost demonstrated strong discriminative ability for predicting CIN2. However, for CIN3, despite maintaining high sensitivity, the model’s low specificity and modest AUC value constrained its effectiveness as a standalone diagnostic tool.

The use of unsupervised clustering identified distinct patient phenotypes linked to different levels of cervical cancer risk. The clusters characterized by age, BMI, sexual behavior, and virological profiles may contribute to the development of personalized risk stratification methods in the future.

Additional validation in prospective cohorts and the integration of longitudinal follow-up data will be essential to assessing their role in informing personalized prevention strategies for cervical cancer. Moreover, future work should investigate whether the integration of unsupervised clustering assignments into predictive models can further enhance accuracy and patient-specific risk stratification in the context of cervical dysplasia.

## Figures and Tables

**Table 1:** Clinical characteristics of evaluated patients depending on the CINTEC^®^ PLUS test results.

Variable	CINtec^®^ PLUS Negative (63 Patients)	CINtec^®^ PLUS Positive (71 Patients)	*p*-Value
Age, years (mean and standard deviation)	35.63 ± 9.52	36.58 ± 9.70	0.572
BMI, kg/m^2^ (mean and standard deviation)	24.16 ± 4.03	22.17 ± 3.25	0.001
Residence (n/%)	Urban = 40 (63.49%)	Urban = 43 (60.56%)	0.727
	Rural = 23 (36.51%)	Rural = 28 (39.44%)	
Smoking history (n/%)	7 (11.11%)	25 (35.21%)	0.001
Multiple sexual partners (n/%)	4 (6.35%)	7 (9.86%)	0.46
Early sexual debut (n/%)	5 (7.94%)	6 (8.45%)	0.914
Use of oral contraceptives (n/%)	9 (14.29%)	12 (16.90%)	0.678
Nulliparity (n/%)	24 (38.10%)	31 (43.66%)	0.566
Sexually transmitted infections (n/%)	0 (0.00%)	4 (5.63%)	0.056
HPV vaccination history (n/%)	1 (1.59%)	4 (5.63%)	0.217
HPV16/18 (n/%)	15 (23.81%)	27 (38.03%)	0.077
Other HR HPV (n/%)	31 (50.00%)	41 (57.75%)	0.371
LR HPV (n/%)	11 (18.03%)	10 (14.08%)	0.536
HSIL (Cytology) (n/%)	4 (6.35%)	23 (32.39%)	<0.001
ASC-US (Cytology) (n/%)	39 (61.90%)	49 (69.01%)	0.387
ASC-US (Cytology) (n/%)	24 (38.10%)	22 (30.99%)	0.387
NILM (Cytology) (n/%)	6 (9.52%)	2 (2.82%)	0.102

Legend: BMI—body mass index, HPV—human papillomavirus, HR HPV—high-risk human papillomavirus, LR HPV—low-risk human papillomavirus, HSIL—high-grade squamous intraepithelial lesion, ASC-US—atypical squamous cells of undetermined significance, NILM—negative for intraepithelial lesion or malignancy, CINtec® PLUS—result of the CINTEC® PLUS dual staining test (p16/Ki-67).

**Table 2:** Logistic regression analysis that quantified the impact of various predictors over a positive CINTEC^®^ PLUS test result.

Variable	Odds Ratio	*p*-Value	95% Confidence Interval
Age	1.058	0.027	1.006–1.111
BMI	1.829	0.004	0.729–2.942
Smoking	1.241	0.012	0.079–2.735
Sexual Partners	0.501	0.372	0.110–2.283
Early Sexual Debut	1.110	0.895	0.235–5.249
COC Use	0.595	0.378	0.188–1.888
HPV16/18	0.476	0.127	0.184–1.235
Other HR-HPV	0.610	0.261	0.258–1.442
LSIL	4.967	0.096	0.752–32.793
HSIL	7.06	0.001	2.287–21.836
ASC-US	0.72	0.388	0.356–1.491

Legend: BMI—body mass index, HPV—human papillomavirus, HR HPV—high-risk human papillomavirus, LR HPV—low-risk human papillomavirus, HSIL—high-grade squamous intraepithelial lesion, ASC-US—atypical squamous cells of undetermined significance, COC—combined oral contraceptive.

**Table 3:** Results from ANOVA analysis and post hoc Bonferroni test that evaluated the mean risk scores for a positive CINTEC^®^ PLUS test result across three different risk groups.

Risk Category	Mean	SD	*p*-Value
Low Risk (38 patients)	2.03	0.94	<0.001
Medium Risk (48 patients)	4.98	0.79	
High Risk (47 patients)	8.38	0.99	
Comparison	Mean Difference		*p*-value
Medium Risk vs. Low Risk	2.95		<0.001
High Risk vs. Low Risk	6.36		<0.001
High Risk vs. Medium Risk	3.40		<0.001

Legend: SD—standard deviation.

**Table 4:** Results from ROC analysis that the predictive performance of a positive CINTEC^®^ PLUS for the prediction of CIN2 and CIN3.

Predictor	Outcome	Sensitivity	Specificity	Precision	Accuracy	F1 Score	AUC
Positive CINtec^®^ PLUS	CIN2	0.84	0.63	0.54	0.70	0.66	0.74
Positive CINtec^®^ PLUS	CIN3	0.89	0.50	0.11	0.52	0.20	0.69
XGBoost complete model	CIN2	0.71	0.85	0.71	0.80	0.71	0.90
XGBoost complete model	CIN3	0.96	0.25	0.88	0.85	0.92	0.58

Legend: CIN—cervical intraepithelial neoplasia, AUC—area under the curve.

## Data Availability

The datasets are available from the correspondent authors upon reasonable request due to local policies.

## Abbreviations

The following abbreviations are used in this manuscript:

ANOVA	Analysis of Variance
ASC-US	Atypical Squamous Cells of Undetermined Significance
AUC	Area Under the Curve
BMI	Body Mass Index
CI	Confidence Interval
CIN	Cervical Intraepithelial Neoplasia
CINtec^®^ PLUS	Dual staining test for p16/Ki-67
COC	Combined Oral Contraceptives
DBSCAN	Density-Based Spatial Clustering of Applications with Noise
HPV	Human Papillomavirus
HR-HPV	High-Risk Human Papillomavirus
HSIL	High-Grade Squamous Intraepithelial Lesion
LMR	Lymphocyte-to-Monocyte Ratio
LR-HPV	Low-Risk Human Papillomavirus
LSIL	Low-Grade Squamous Intraepithelial Lesion
MLP	Multilayer Perceptron
NB	Naïve Bayes
NILM	Negative for Intraepithelial Lesion or Malignancy
NLR	Neutrophil-to-Lymphocyte Ratio
NPV	Negative Predictive Value
OR	Odds Ratio
PCA	Principal Component Analysis
PLR	Platelet-to-Lymphocyte Ratio
PPV	Positive Predictive Value
RF	Random Forest
ROC	Receiver Operating Characteristic
SD	Standard Deviation
SHAP	SHapley Additive exPlanations
SMOTE	Synthetic Minority Over-sampling Technique
STIs	Sexually Transmitted Infections
SVM	Support Vector Machine
TCT	ThinPrep Cytologic Test
UMAP	Uniform Manifold Approximation and Projection
XGBoost	eXtreme Gradient Boosting
